# Comparative safety and effectiveness of cryoballoon versus radiofrequency ablation for atrial fibrillation: a systematic review and meta-analysis

**DOI:** 10.1186/s43044-025-00611-9

**Published:** 2025-02-03

**Authors:** Muhammad Furqan, Ifrah Inbisat Raza, Shaheera Younus, Hareer Fatima, Hiba Azhar, Sania Kaneez Fatima, Laiba Ali, Sara Khan, Aayat Ellahi

**Affiliations:** 1 Department of Internal Medicine, Student of Medicine, King Edward Medical University, Neela Gumbad, Nila Gumbad Chowk, Lahore, 54000 Punjab Pakistan; 2 Department of Medicine, Student of Medicine, Jinnah Sindh Medical University, Karachi, Pakistan

**Keywords:** Atrial fibrillation, Paroxysmal atrial fibrillation, Pulmonary vein isolation, Cryoballoon, Radiofrequency ablation

## Abstract

**Background:**

Over the past fifty years, the incidence of atrial fibrillation (AF) has tripled. Traditionally, the main treatment for this condition has been pulmonary vein isolation (PVI) performed using radiofrequency catheter ablation (RFCA). However, another technique known as cryoballoon ablation (CBA) has been developed as another option for managing this heart rhythm disorder. This study evaluated the efficacy and safety of CBA and RFCA for the treatment of AF.

**Methods:**

This study compared the safety and effectiveness of CBA and RFCA for the treatment of AF using a thorough review of randomized controlled trials up until June 1, 2023.

**Results:**

The results revealed that CBA and RFCA had similar effectiveness and safety profiles in achieving freedom from AF (RR: 1.00; 95% CI: 0.93 to 1.07, p = 0.99) and paroxysmal atrial fibrillation (PAF) (RR: 0.99; 95% CI: 0.89 to 1.10, p = 0.79). CBA was faster (MD =  − 23.99; 95% CI: − 32.98 to − 15.00; p < 0.00001) with a higher risk of phrenic nerve palsy (RR = 6.88; 95% CI: 3.26 to 14.50, p < 0.00001). Acute PVI rate (RR = 1.0; 95% CI: 0.99 to 1.01, p = 0.95), overall complications (RR = 1.37; 95% CI: 0.93 to 2.01, p = 0.11), pericardial effusion (RR = 0.59; 95% CI: 0.25 to 1.41, p = 0.24), and fluoroscopy time (MD = 1.63; 95% CI: − 2.06 5.32; p = 0.39) did not significantly differ between the two procedures.

**Conclusions:**

CBA and RFCA offer similar outcomes for patients with AF and PAF, with CBA being quicker but carrying a slightly higher risk of phrenic nerve palsy.

**Supplementary Information:**

The online version contains supplementary material available at 10.1186/s43044-025-00611-9.

## Background

The most common kind of cardiac arrhythmia, atrial fibrillation, is characterized by erratic electrical impulses in the atria. This irregularity causes the atria to quiver or “fibrillate” [1]. Classified as a tachyarrhythmia, atrial fibrillation typically results in an accelerated heartbeat. Episodes can be brief, lasting less than seven days (termed paroxysmal), or they can persist for longer than seven days (called persistent). The range of symptoms associated with this condition can be broad: some individuals may experience no symptoms at all, while others might encounter chest discomfort, palpitations, elevated heart rate, breathlessness, nausea, dizziness, excessive sweating, or overwhelming tiredness [1]. Additionally, there is a connection between AF and an elevated risk of heart failure, stroke, and death [2]. The prevalence of AF has risen over the last 50 years, and as of 2016, 46.3 million people worldwide were thought to be affected by the illness. In the United States (U.S.) alone, approximately 3 to 6 million individuals suffer from it, and by 2050, it is estimated that the burden will increase to approximately 6 to 16 million [3].

The pathophysiology of AF involves the rapid ectopic firing of impulses that arise from myocyte sleeves within the pulmonary veins and results in propagating reentrant waves in vulnerable atrial tissue [4]. Thus, the cornerstone of interventional methods for treating AF is pulmonary vein isolation (PVI) ablation, particularly when patients are not responsive to medication [5].

PVI with radiofrequency catheter ablation (RFCA) is the most common ablation procedure performed nowadays for treating AF and utilizes radiofrequency waves to create a circumferential lesion that isolates the PV from the left atrium [6]. However, it offers disadvantages such as longer procedural time and operator dependency [7]. To overcome these procedural difficulties, cryoballoon ablation (CBA) has been developed, which, unlike RFCA, uses a single catheter to deliver ablative cryo-energy at the PV antrum and thus amounts to less time consumption. However, this treatment approach is associated with an increased incidence of recurrence, particularly in cases of persistent atrial fibrillation [8]. However, when comparing the overall safety of the two ablation techniques, no significant difference was observed [9].

Despite comprehensive research in the field of catheter ablation for the past many years, recurrence rates of AF after a single PVI procedure remain at about 10–25% [10]. Additionally, the currently available data comparing RFCA to CBA show variable results. Some studies suggest that RFCA may have a safer profile, noting fewer nerve injuries compared to CBA. [11]. Conversely, other research indicates that there are no significant statistical differences in safety between these two ablation techniques [12]. For this reason, it remains a matter of ongoing debate whether CBA is a valuable or better alternative to RFCA.

Assuming that the disparity is likely due to the heterogeneous selection of patients, small sample sizes, and variable follow-up durations, we conducted a meta-analysis. The goal of this study is to compare and thoroughly assess the safety and efficacy of RFCA against (CBA) in treating AF, both paroxysmal and persistent.

## Methods

### Data sources and search strategy

This analysis follows the guidelines outlined by the “Preferred Reporting Items for Systematic Reviews and Meta-Analyses” (PRISMA), ensuring adherence to standardized reporting practices [13]. To comprehensively gather relevant studies, a thorough search was conducted in major databases including MEDLINE (via PubMed), Embase, Scopus, and the Cochrane Library. The search encompassed studies published until June 1, 2023, aiming to include a broad spectrum of systematic reviews and meta-analyses for inclusion in the analysis. Our goal in utilizing both datasets was to reduce the possibility of publication bias. To find papers pertinent to our investigation, we employed a carefully crafted search string in our search approach. The search string was constructed using diverse keywords such as “atrial fibrillation”, “AF”, “radiofrequency ablation”, “cryoablation”, “cryoballoon”, and “pulmonary vein isolation”, enabling the manual retrieval and subsequent assessment of relevant articles (Supplemental Table S1).

### Inclusion criteria

The studies that qualified for inclusion in this meta-analysis fulfilled the following conditions: (1) RCTs published up to June 1, 2023. (2) A population of more than 60 participants undergoing PVI. (3) Comparison between cryoballoon and radiofrequency ablation. (4) Patients were free from AF in both groups as the primary outcome. (5) Follow-up duration > 6 weeks.

### Data extraction and quality assessment

The initial phase of screening involved reviewing titles and abstracts to identify studies that did not align with our inclusion criteria. We employed EndNote Reference Library software to manage references and prevent duplication of articles. Subsequently, full-text versions of the articles were obtained and carefully reviewed to assess their appropriateness for inclusion in the meta-analysis. Four researchers (L.A., M.F., H.A., and I.R.) participated in the data extraction process from each RCT that met our criteria. To ensure the reliability of our data extraction process, three authors conducted simultaneous extractions following strictly predefined eligibility criteria. Subsequently, a fourth author independently cross-verified all data extracted from the included studies. Any discrepancies or disagreements between the authors were thoroughly discussed and resolved through a consensus approach. This systematic procedure was implemented to strengthen the internal validity and consistency of data extraction.

The information gathered from each study included initial participant demographics, specifics of the interventions administered, and the results obtained. The primary outcome of interest in our meta-analysis was recovery from atrial fibrillation, and the secondary outcomes of interest were total complications, transient phrenic nerve palsy, pericardial effusion, procedure time, fluoroscopy time, and effectiveness of acute PVI. Any discrepancies or uncertainties during the data extraction process were resolved through collaborative discussions among the authors. To evaluate the integrity of the included studies, one of our authors, M.F., employed the Cochrane risk-of-bias tool for randomized trials (RoB 2) to determine the potential bias in each study under review [14].

### Statistical analysis

In our meta-analysis, we utilized the Review Manager Software (RevMan 5.4.1, Cochrane Collaboration, 2020) to carry out our statistical analyses. We computed risk ratios (RRs) and their 95% confidence intervals (CIs) to evaluate the significance of differences observed between the CBA and RFCA study groups. The selection of either a fixed-effects or a random-effects statistical model was dependent on whether the studies demonstrated homogeneity or heterogeneity, respectively. This determination of homogeneity versus heterogeneity was based on the I2 statistic. We considered results statistically significant if they yielded a P-value of less than 0.05, using a two-tailed test.

## Results

### Studies selection

**Fig. 1:**
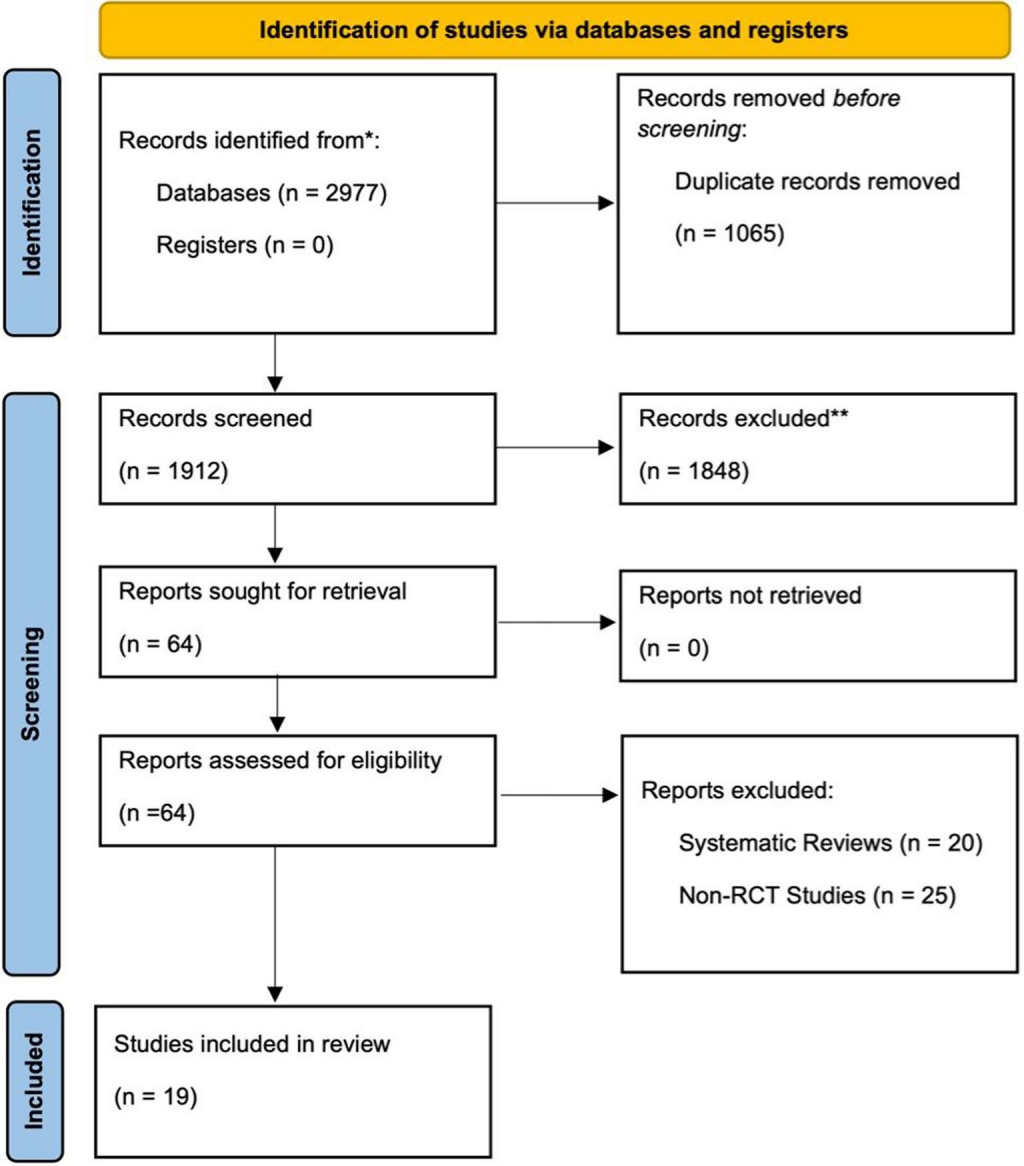
Flow diagram of study selection. RCT = Randomized Control Trial

Figure 1 illustrates the study selection process. Our initial database search yielded 2977 articles, from which 1065 duplicates were subsequently removed, resulting in 1912 potentially relevant articles. After a detailed review of titles and abstracts, 1848 articles were excluded due to irrelevance to the research topic, leaving 64 articles for more in-depth evaluation. Upon further scrutiny, 45 were excluded, comprising 20 systematic reviews and 25 non-randomized controlled trials (RCTs). Consequently, 19 RCTs [9–11, 15–30] were included in the meta-analysis. Notably, all included RCTs utilized either the first-generation Arctic Front Cryoablation System or second-generation Arctic Front Advance Cryoballoon System for their interventions. Seven studies examined repeat procedures for AF ablation, including cases of repeat ablation for recurrent arrhythmias, symptomatic atrial tachyarrhythmias, failed previous procedures, and AF recurrence after 4 to 6 months [10, 11, 17, 19, 21, 24, 26].

Various studies have employed repeat procedures for AF ablation, utilizing methods such as PV re-isolation with substrate modification or electrogram-guided ablation [10], ablative procedures for recurrences, linear radiofrequency (RF) ablation in the left atrium (LA) [17], and with catheters and electroanatomical mapping [21, 26]. Some studies used approaches consistent with the initial PV isolation methods, whether through RFCA or CBA, addressing recurrent arrhythmias and symptomatic atrial tachyarrhythmias [11, 19, 24].

### Baseline characteristics of included studies

Table 1 presents the foundational characteristics of the studies included in the analysis. A cumulative total of 3,388 participants were assessed across these studies. Of these participants, a majority, amounting to 2,162 individuals or 63.8%, were male. Within this population, an average of 4.5% had diabetes mellitus in each group, and 3% in each group were hypertensive. In addition, the mean LAD for the CB ablation group was 41.16 mm and RF ablation was 41.45 mm. People who participated were also followed up for an average of 13.48 months.

**Table 1:** Baseline Characteristics of 19 Included Randomized Controlled Trials

Study	Patients, n CB/RF	Mean age, years CB/RF	N (MALES, %) CB/RF	STUDY POPULATION, CB/RF	Diabetes mellitus, % CB/RF	Hypertension, % CB/RF	CAD, % CB/RF	LVEF %, CB/RF	MEAN LAD, mm, CB/RF	Follow up, Months
Herrera et al. (2012)	30/30	57/56	25 (83.3)/23 (76.7)	PAF21 (70%) PAF17 (56.7%)	NR	13 (43.3%)/14 (46.7%)	3 (10.0%)/4 (13.3%)	NR	41.1/40.0	12
Pokushalov et al. (2013)	40/40	56/56	31 (77.5)/33 (82.5)	PAF	2 (5%)/3 (7%)	6 (15.0%)/7 (17.0%)	NR	58/57	46/48	12
Malmborg et al. (2013)	54/56	59/62	43 (79.6)/40 (71.4)	PAF39 (72.2%)/ PAF37 (66.1%)	NR	22 (40.7%)/35 (62.5%)	4 (7.4%)/6 (10.7%)	NR	40/42	12
Perez-Castellano et al. (2014)	25/25	58/56	17 (68)/22 (88)	PAF	4 (16%)/2 (8%)	6 (24)/8 (32)	NR	NR	42/42	12
Luik et al. (2015)	156/159	61/60	100 (64.1)/91 (57.2)	PAF	14 (9%)/17 (10.7%)	96 (62.3%)/103 (66%)	19 (12.2%)/20 (12.6%)	NR	NR	12
Hunter et al. (2015)	78/77	56/61	56 (72)/47 (61)	PAF	4 (5%)/5 (6%)	27 (35%)/23 (30%)	6 (8%)/6 (8%)	NR	42/43	12
Kuck et al. (2016)	374/376	59.9/60.1	221 (59)/236 (63)	PAF	37 (9.9%)/22 (5.9%)	215 (57.5%)/221 (58.8%)	31 (8.3%)/32 (8.5%)	NR	40.2/40.6	18
R et al. (2018)	67/67	55.7/60.7	48 (71.6)/42 (62.7)	PAF	4 (6%)/5 (7.5%)	25 (37.3%)/21 (31.3%)	NR	NR	42.4/43	18
Buist et al. (2018)	133/136	59.7/58.2	92 (69.2)/99 (72.8)	PAF117 (88%)/PAF112 (82.4%)	14 (10.6%)/9 (16.7%)	57 (43.2%)/52 (38.5%)	4 (7.4%)/6 (10.7%)	NR	NR	12
Gunawardene et al. (2018)	30/30	62/57.4	18 (60)/24 (80)	PAF	NR	16 (53%)/17 (56%)	NR	59.8/59.2	NR	10.3
Davtyan et al. (2018)	45/44	57.6/55.6	22 (48.9)/19 (43.2)	PAF	2 (4.4%)/6 (13.6%)	35 (77.8%)/34 (77.3%)	4 (8.9%)/2 (4.5%)	NR	41.1/40.0	12
Andrade et al. (2019)	231/115	58.9/58.6	152 (65.8)/79 (68.7)	PAF222 (96.1%)PAF105(91.3%)	NR	80 (34.6%)/40 (34.8%)	19 (8.2%)/6 (5.2%)	59.3/59.1	38.0/37.4	12
Giannopoulos et al. (2019)	80/40	61/58	NR	PAF	9 (11.3%)/6 (15%)	41 (51.3%)/18 (45%)	6 (7.5%)/2 (5.0%)	60/60	40/41.5	6
Sorensen et al. (2021)	49/49	60/62	32 (65)/35 (71)	PAF	NR	14 (29%)/13 (27%)	7 (14%)/3 (6%)	NR	40.2/40.4	12
Shi et al. (2022)	52/49	62.4/64	45 (86.5)/35 (71.4)	Persistent AF	1(1.9%)/4(8.2%)	29 (55.8%)/28 (58%)	4 (7.7%)/6 (12.2%)	56/56.8	46/44	12
Baimbetov et al. (2022)	50/50	61.3/61.6	31(62%) / 29 (58%)	PAF	NR	NR	NR	59/54	41/39	36
Theis et al. (2021)	75/75	61.6/66.2	41 (55)/45 (60)	PAF	NR	44 (59%)/55 (73%)	6 (8%)/10 (13.3%)	55.7/56.4	39/41	12
Mililis et al. (2023)	66/133	62.7/60.2	51(77.3)/111(83.3)	Persistent AF	9(13.6%)/20(15%)	NR	NR	55.7/55.1	43.6/44.9	12
Yan et al. (2023)	92/138	59.9/60	48(52.1%)/79(57.25%)	PAF	7(7.61%)/21(15.22%	33(35.87%)/59(42.75%)	7(7.61%)/9(6.52%)	68.1/66.8	36.1/36.5	12

### Quality assessment

**Fig. 2:**
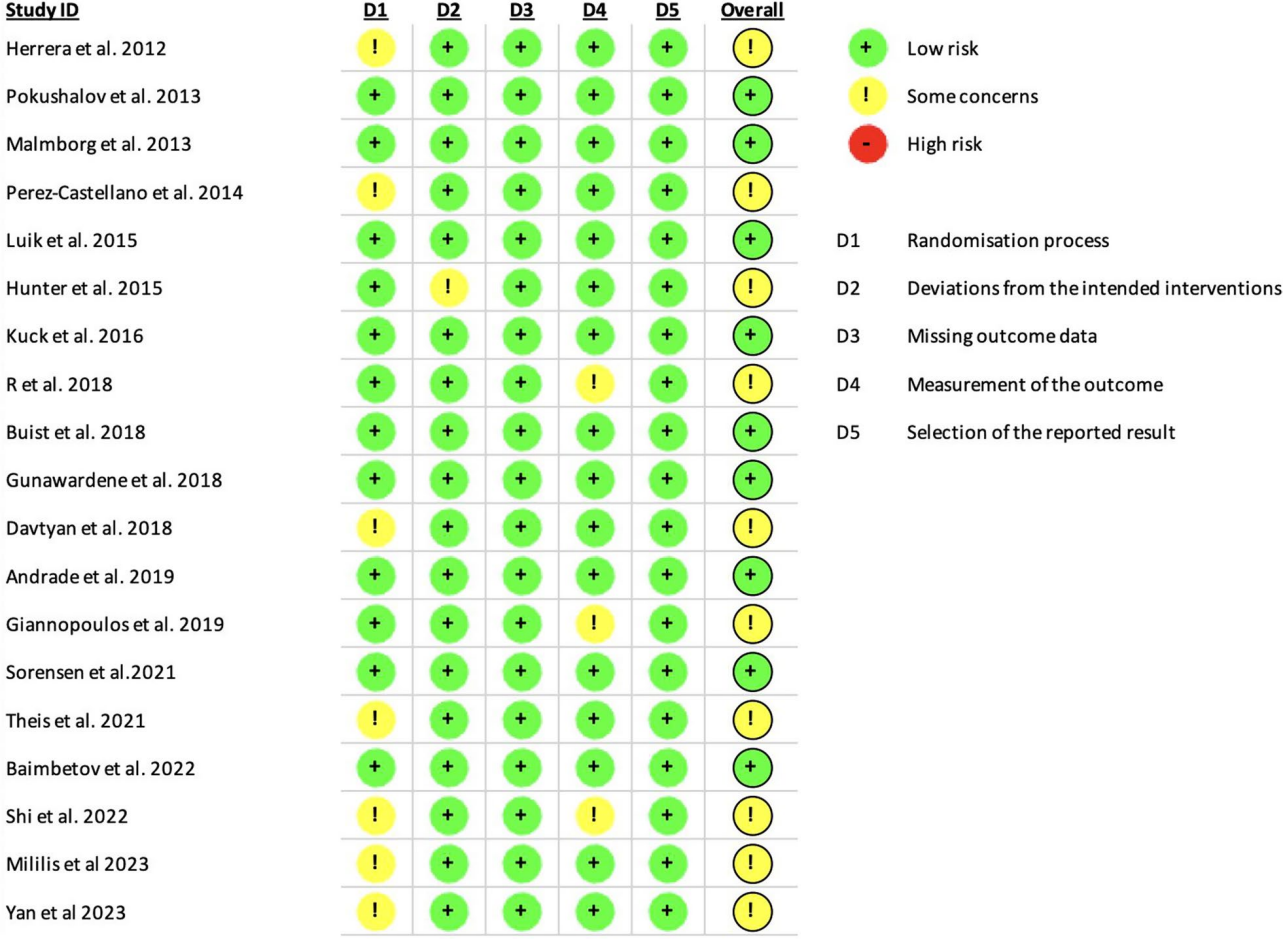
Quality judgments about each risk of bias item

In this review, the Cochrane Risk of Bias 2 tool was applied to assess the integrity of the included studies, with findings displayed in Figure 2. The assessment revealed that nine of the studies were determined to be of high quality, exhibiting a low risk of bias and thereby supporting their credibility. However, ten of the studies displayed indications of bias, mainly due to shortcomings in their randomization methods.

### Effects of intervention

#### Primary endpoints

##### Freedom from AF in patients

**Fig. 3:**
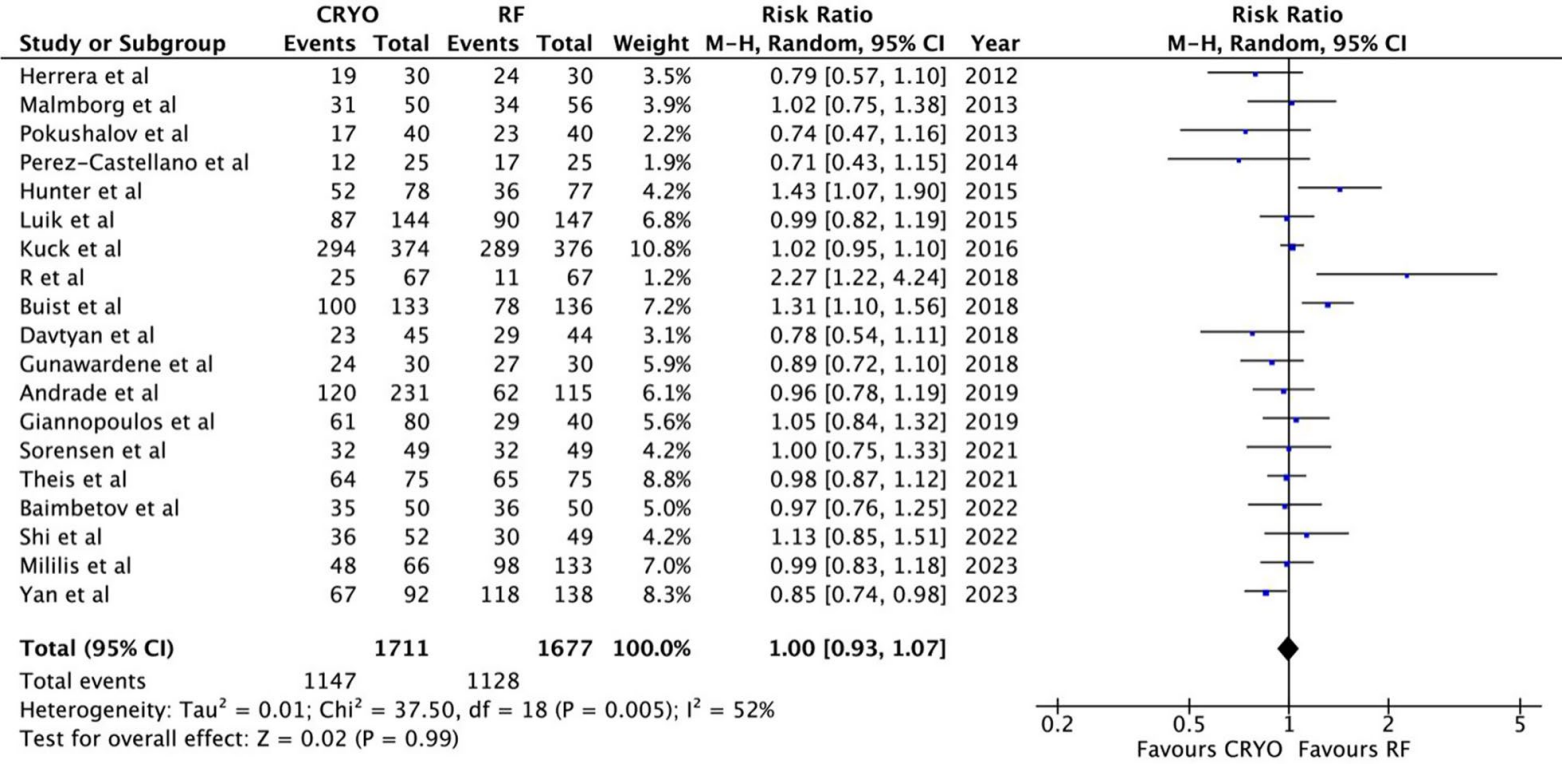
Forest plot for the proportion of patients free from AF in all 19 studies. AF indicates atrial fibrillation

A comprehensive analysis of 19 studies involving 3416 participants examined the primary endpoint of achieving freedom from AF. The investigation compared two groups undergoing different treatment modalities: cryoballoon ablation (CBA) and radiofrequency catheter ablation (RFCA). The findings indicated a similar success rate in both groups, with 67% (1,147 individuals) in the CBA group and 67.3% (1,128 individuals) in the RFCA group reporting freedom from AF. A pooled analysis of the data confirmed that there was no statistically significant difference in the outcomes between the two treatment options regarding their effectiveness in achieving freedom from AF. (RR: 1.00; 95% CI: 0.93 to 1.07, p = 0.99). However, heterogeneity was observed among these studies, indicating some variations in the results (I2 = 52%, p = 0.005) (Fig. 3).

We performed a sensitivity analysis to address the heterogeneity in the outcomes. After removing Hunter et al. [19], Buist et al. [21], and R et al. [20], the results were still consistent (RR = 1.00; 95% CI: 0.93–1.07; p = 0.99), but the heterogeneity decreased to I2 = 0%. (Supplemental Figure S7).

##### Freedom from AF in patients with paroxysmal atrial fibrillation (PAF)

**Fig. 4:**
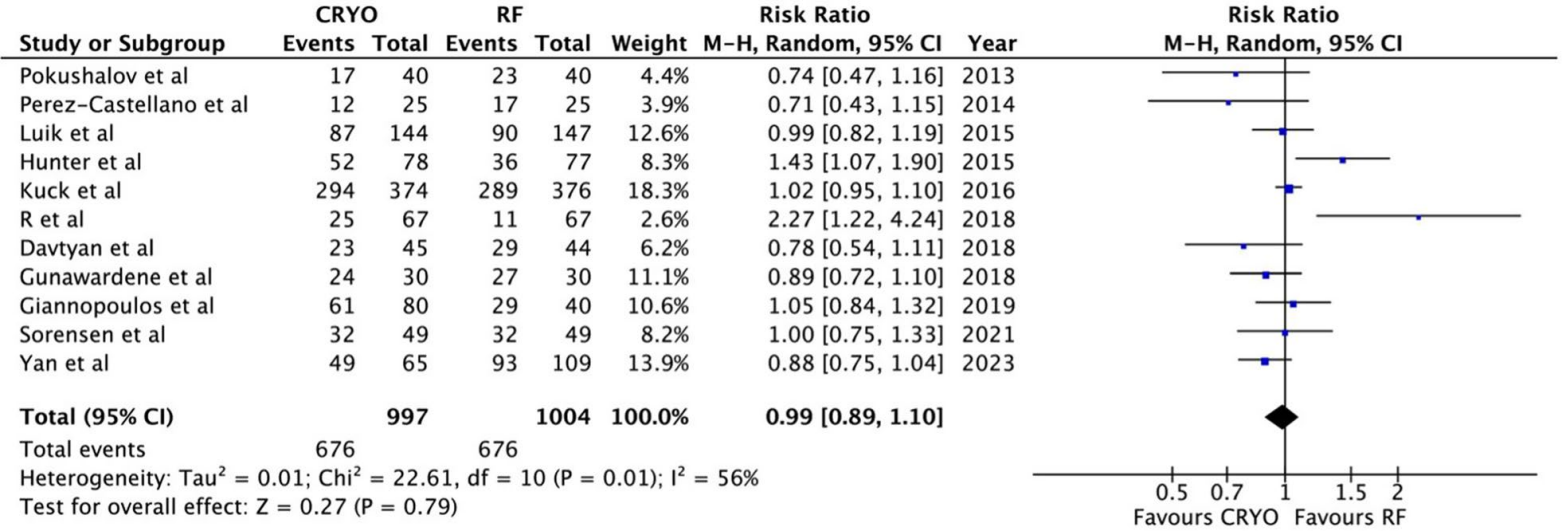
Forest plot for the proportion of patients free from AF in 11 studies only containing participants with paroxysmal AF. AF indicates atrial fibrillation

Among the studies analyzed, 11 specifically examined patients with PAF. When these studies were examined more closely in a subgroup analysis, it was found that there was no notable difference in success rates between the two treatment groups. Specifically, 67.8% of patients treated with CBA achieved freedom from AF, compared to 67.3% of those who underwent RFCA. This indicates that both treatments were similarly effective for this patient subgroup (RR: 0.99; 95% CI: 0.89 to 1.10, p = 0.79; I2 = 56%, p = 0.01). (Fig. 4).

Sensitivity analysis was also performed for this result. Upon removing Hunter et al. [19] and R et al. [20], the heterogeneity lowered to I2 = 0, while the results remained consistent (RR = 0.99; 95% CI: 0.89–1.10; p = 0.79; p = 0.01). (Supplemental Figure S8).

#### Secondary endpoints

##### Acute pulmonary vein isolation (PVI)

A comprehensive analysis of fourteen studies investigating the efficacy of PVI procedures. The analysis revealed that the success rate of achieving immediate PVI in patients undergoing CBA stood at 97.9%, based on a cohort of 1311 individuals. Conversely, those undergoing RFCA achieved a slightly higher success rate of 98.5% among 1,236 patients. However, when subjected to statistical scrutiny, no notable disparities in the success rates of achieving acute PVI emerged between the two treatment modalities (RR = 1.0; 95% CI: 0.99 to 1.01, p = 0.95; I2 = 56%). (Supplemental Figure S1).

##### Overall complications

In a comprehensive analysis focused on the complications associated with specific medical procedures, researchers examined data from 15 different studies. They investigated the incidence of complications among patients, noting that 5.7% of patients (79 individuals) who underwent radiofrequency catheter ablation (RFCA) experienced complications. Similarly, 7.6% of patients (110 individuals) treated with cryoballoon ablation (CBA) also faced complications. The findings from this analysis revealed no statistically significant differences in the rates of complications between the RFCA and CBA patient groups (RR = 1.37; 95% CI: 0.93 to 2.01, p = 0.11; I2 = 22%). (Supplemental Figure S2).

##### Pericardial effusion

A review of data from 16 different studies indicated that in the group undergoing RFCA, pericardial effusion occurred in 0.9% of cases, affecting 14 individuals. In contrast, among those treated with CBA, the incidence was slightly lower at 0.46%, involving 7 patients. Statistical analysis showed that the difference in the occurrence of pericardial effusion between the RFCA and CBA groups was not significant, suggesting that both treatments have comparable safety profiles concerning this specific complication (RR = 0.59; 95% CI: 0.25 to 1.41, p = 0.24; I2 = 0%). (Supplemental Figure S3).

##### Transient phrenic nerve palsy

A review of 17 studies investigating the occurrence of transient phrenic nerve palsy revealed that this side effect was more commonly seen in patients undergoing CBA. Specifically, about 3.64% of patients in the CBA group, which translates to 57 individuals, experienced this complication. On the other hand, RFCA showed a much lower occurrence rate, with only one reported case amounting to a 0.1% incidence rate. The data robustly suggests that there is a significantly higher risk of developing transient phrenic nerve palsy with CBA procedures compared to RFCA (RR = 6.88; 95% CI: 3.26 to 14.50, p < 0.00001; I2 = 0%). (Supplemental Figure S4). Resolution of phrenic nerve paralysis varied significantly across the studies included in this meta-analysis. Many of them were completely resolved within 24 hours of the beginning of the procedure [16, 17, 22, 27], while some took about one week for complete resolution [17, 27]. The majority recovered within 6 months [9, 23, 24, 26–28]. One study reported an average recovery period of 8.5 months for 4 cases in total [19]; meanwhile, only one case reported unresolved nerve palsy even at the end of 12 months, post-procedure [9].

##### Procedure time

An analysis of data collected from 16 studies was conducted to evaluate the duration of procedures across two different groups. Findings from this analysis revealed that the group undergoing CBA experienced notably shorter procedure times compared to the group treated with RFCA (mean difference [MD] = −23.99; 95% CI: −32.98 to −15.00; p < 0.00001; I2 = 93%). (Supplemental Figure S5).

##### Fluoroscopy time

An analysis involving fifteen studies assessed the duration of fluoroscopy and found no statistically significant differences between the two groups examined (MD = 1.63; 95% CI: −2.06 to 5.32; p = 0.39; I2 = 97%). (Supplemental Figure S6).

### Publication bias

**Fig. 5:**
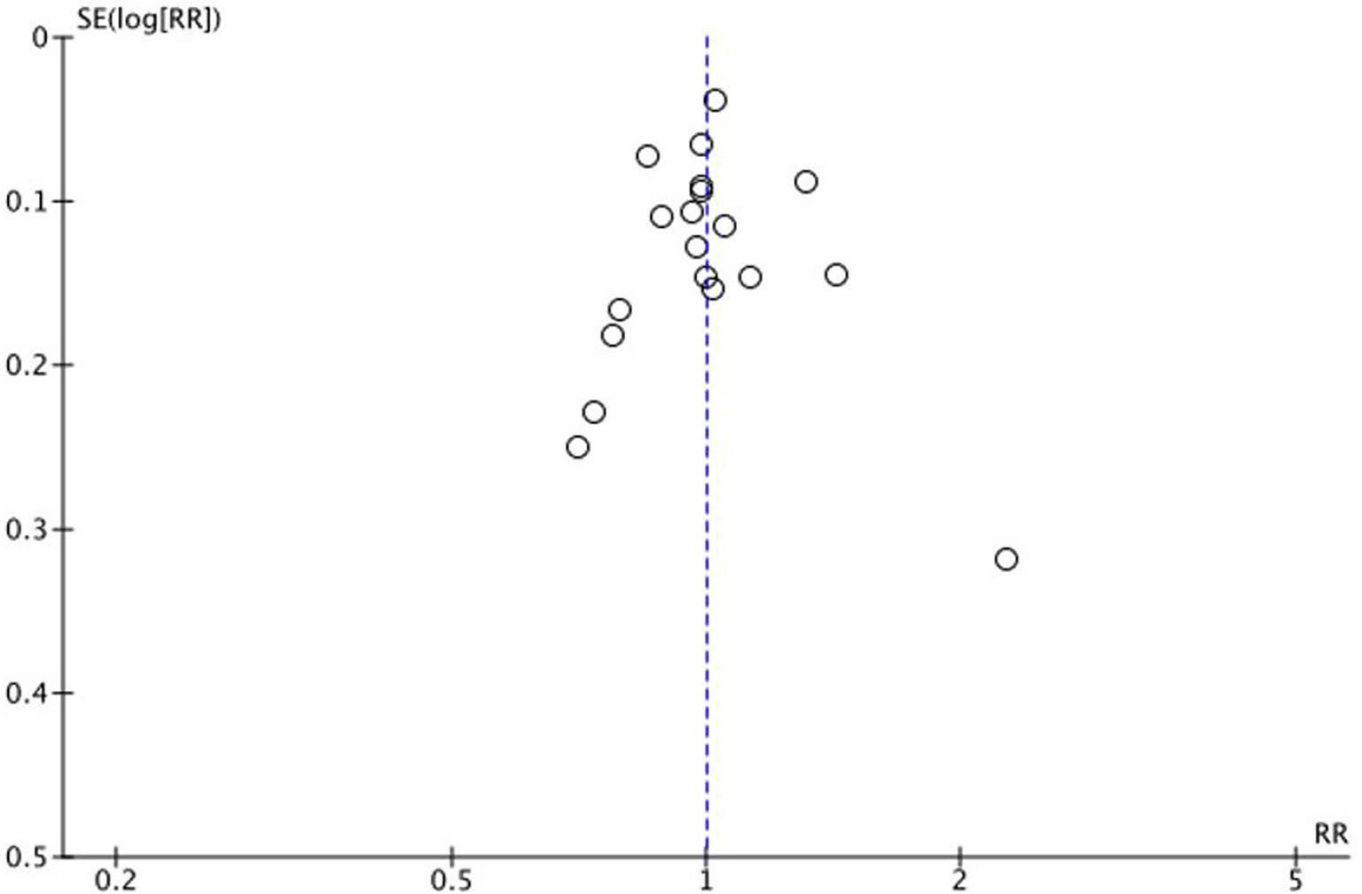
The funnel plot for the proportion of patients free from AF. AF indicates atrial fibrillation

Upon reviewing the funnel plot that incorporates data from 19 different studies evaluating the percentage of patients who remained free from AF, it was observed that the distribution of studies within the plot was symmetrical. This symmetry in the distribution typically suggests a low likelihood of publication bias influencing the results. Moreover, the concentration of studies near the top end of the plot indicates that the impact of any potential publication bias among these studies is probably negligible. This finding is important as it supports the reliability of the study conclusions regarding the proportion of patients staying free from AF (Fig. 5).

## Discussion

In this comprehensive meta-analysis which included 3,380 individuals diagnosed with AF, there is robust support for the use of RFCA and CBA as treatment options. The main results from this study indicate that both RFCA and CBA are equally safe and effective for managing atrial fibrillation, including both persistent and paroxysmal forms of the condition. When considering the secondary outcomes, we found no difference in acute PVI, overall complications, occurrence of pericardial effusion, and fluoroscopy time in both groups. However, the CBA group exhibited a shorter procedural time and a heightened occurrence of temporary phrenic nerve palsy when compared with the RFCA group.

Our meta-analysis findings are consistent with those of other recent meta-analyses, and we also identified some significant distinctions between our meta-analysis and those of other researchers in the area. For example, in their meta-analysis involving a significantly larger sample, Cardoso et al. [31] reported their results without revealing significant insights into the efficacy of PVI, or the durations of the procedure and fluoroscopy used. Chen et al. [32] included both RCTs and non-RCTs, leading to a sample size larger than our meta-analysis. However, the sample analyzed was limited to patients presenting with paroxysmal atrial fibrillation (PAF), whereas our meta-analysis included both paroxysmal and persistent forms of the condition. Furthermore, significant outcome measures, such as the number of patients remaining free from AF, PVI effectiveness, the occurrence of pericardial effusion as a complication, and duration of fluoroscopy and the overall procedure, were not addressed. Chen et al. [33] carried out another meta-analysis that included RCTs and other cohort studies and was limited to patients with PAF and second-generation cryoballoon treatments. These results are different those from of our meta-analysis because their meta-analysis showed a high free AF period due to cryoballoon. The lack of participants with chronic atrial fibrillation, coupled with the presence of cohort studies and a substantial number of subjects may account for the observed results. In a study by Wu et al. [34], a meta-analysis compared the effectiveness of two different types of ablation techniques: second-generation cryoballoon ablation (CBA-2G) and contact force radiofrequency ablation (RFA-CF). Their analysis, which included six randomized controlled trials (RCTs), found no significant differences in effectiveness between the two methods. Notably, Wu et al. also reported a consistent primary outcome across studies, with less variability (I2 = 52%) than what we observed in our research. This suggests a potential contrast in findings due to differences in study size or methodology, as Wu et al.’s study had a relatively small sample. In another comparison, Ravi et al. [35] found that CBA-2G typically required longer fluoroscopy times than RFA-CF, which contrasts with our findings where fluoroscopy durations did not differ significantly between the CBA and RF treatment groups. This discrepancy highlights the variability in outcomes that can arise from different patient groups or procedural specifics. Despite the overall findings being consistent with previous studies, we also observed considerable heterogeneity among the primary outcomes: freedom from AF in all patients (I2 = 52%) and in patients with PAF (I2 = 56%) in our study. To address the heterogeneity, we performed a sensitivity analysis which substantially lowered the heterogeneity (I2 = 0%). (Supplemental Figures S7, S8).

The landmark trial FIRE AND ICE has also proven CBA as an equally effective alternative to RFCA for the treatment of PAF [36]. A study utilizing a modified intention-to-treat approach showed that individuals treated with CBA were more likely to undergo additional ablations, cardioversions, and hospital readmissions for cardiovascular issues, compared to those who underwent RFCA. Additionally, the analysis indicated that when the initial isolation of the pulmonary veins was performed using CBA, there was a significant decrease in the instances of restored electrical connection between the pulmonary veins and the left atrium during the period of follow-up. The lower stability of RF catheters compared to cryoballoon catheters could potentially be the reason for these results. Moreover, transient phrenic nerve palsy is a known complication associated with CBA. Tohoku et al. [37] also reported this outcome and explained the mechanism of phrenic nerve palsy in CBA that occurs due to electrical dysfunction of the nerve due to thermal injury. The formation of lesions through the cryoballoon involves a process in which cryothermal energy is uniformly transferred from the balloon to the adjacent cardiac endocardium. Achieving successful outcomes with this method requires ensuring that the pulmonary veins are completely occluded, a task that frequently necessitates the use of the catheter-pushing technique. This technique may decrease the distance between the balloon’s surface and the phrenic nerve that runs along the pericardium, thereby raising the risk of damaging the phrenic nerve during a cryoballoon ablation procedure. Such damage can lead to phrenic nerve palsy, a condition affecting nerve function [37].

According to our meta-analysis, the RFCA group’s procedure time was substantially longer than that of the CBA group. The mechanism of RFCA can be explained by the fact that RFCA targets cardiac tissue and causes its cauterization using radiofrequency energy [38]. On the other hand, for CBA, an interatrial septal puncture is performed and a catheter is inserted into the lumen of the cryoballoon for electrogram recording of the PV [39]. The catheters used for CBA are similar in size to the pulmonary vein, which is larger than conventional RFCA catheters. For this reason, they can apply energy in a single application and cover greater areas with a homogenous icecap compared to RFCA catheters that apply energy in multiple applications. This technical advantage of CBA decreases the procedure time significantly, as demonstrated in our analysis [40]. Additionally, left atrial dwell time, the length of time during the procedure in which the catheter stays in the left atrium, was accounted for in two of the 19 studies included in this meta-analysis [9, 27]. In both of these, the LA dwell time, in relation to the complications, was found to be significant (p-value < 0.001). Kuck et al. found that in the RFCA group (N = 376), it was 108.6 ± 44.9 min, compared to the CBA group (N = 374), which reported 92.3 ± 31.4 min, a significantly shorter duration [9]. Similarly, Baimbetov et al. calculated a mean of 93.66 min in the RFCA group (N = 50), while the CBA group (N = 50) reported a significantly shorter mean duration of 72.30 min [27].

These findings indicate that CBA and RF are comparably effective for treating both persistent and paroxysmal atrial fibrillation, with similar rates of patients remaining free of arrhythmias. However, CBA may be preferred when reduced procedural time is a priority. Despite these findings, further research is needed to fully assess the effectiveness of these treatments across various patient groups and to improve techniques that will better patient results and reduce the risks associated with the procedures.

Investigating innovative methods to improve the effectiveness of ablation procedures, such as pulsed-field ablation (PFA), appears to be a promising option for future applications. The PULSED AF Pilot Trial (study identifier: NCT04198701) provided some important data on the use of a PFA system for treating patients with atrial fibrillation. The trial successfully isolated all 152 pulmonary veins in the 38 participants. On average, the entire procedure took about 160 ± 91 min, with approximately 82 ± 35 min spent specifically in the left atrium and around 28 ± 9 min using fluoroscopy. Throughout the 30-day post-procedure monitoring period, the PFA system did not result in any major complications, including damage to the phrenic nerve or esophagus, stroke, or death [41]. However, to fully understand the risks and effectiveness of this treatment, longer-term studies are needed to monitor for any potential delayed effects and to confirm whether the ablation consistently prevents the recurrence of atrial fibrillation over time. Furthermore, ongoing research and efforts focusing on the development of targeted pharmacological treatments for AF are recommended. Clinicians can use these findings to inform their decision-making and select the most appropriate ablation technique based on individual patient characteristics and preferences. Recently, catheter-based electrical isolation of pulmonary veins, (PFA), has gained popularity as a potential novel treatment for AF with a significantly lower complication rate than thermal ablation [42]. Most of the complications related to thermal ablation result from the propagation of heat through tissue and the resulting indiscriminate destruction of nearby structures, for example, atrio-esophageal fistulae which carry a mortality rate of over 50% [43]. Contrary to that, early trials of PFA show selective tissue ablation and essentially no risk of esophageal complications. However, this technique has a significantly higher rate of vascular injuries (for example, cardiac tamponade and local hematomas), which is why further research is necessary to test its safety [42].

### Study limitations

Our meta-analysis included studies conducted over an extended period that included data from inception to June 2023. The sample size was limited in the majority of studies. Furthermore, the majority of the studies reviewed did not provide comprehensive details regarding the expertise and experience levels of the operators involved. A concern that persists is the heterogeneity in how long patients have had AF or the extent of their AF before undergoing ablation. Additionally, only three out of nineteen studies included patients exclusively suffering from persistent AF, while the rest had patients with both persistent AF and PAF enrolled randomly. As a result, this meta-analysis does not offer a thorough assessment of the effects of ablation on patients who specifically have persistent AF. The lack of any detectable arrhythmia following the three-month blanking phase, including atrial tachycardia, atrial flutter, or fibrillation, post-ablation, was referred to as freedom from AF. However, only eight studies recorded atrial arrhythmias lasting longer than two minutes and monitored these recurrences using implanted loop recorders. In contrast, other trials used a 24-h or 7-day Holter monitor to document arrhythmia recurrences and considered episodes lasting > 30 s. The latter could have resulted in some disparity by underestimating AF recurrence. Additionally, the study covers a broad time spectrum, analyzing RFCA as a unified entity despite evolving technologies. This approach, encompassing various technologies, may hinder meaningful conclusions from physicians considering these two technologies. This meta-analysis lacked data on operator experience and procedural volume for RFCA, limiting insights into their impact. This may affect the generalizability of our findings on RFCA versus CBA. Moreover, the follow-up (FU) methodology varied across studies which poses another limitation as variation in detection sensitivity that may influence the results.

## Conclusion

Our meta-analysis demonstrated that CBA and RFCA have the same efficacy and are equally safe in treating patients with AF and PAF. The analysis further revealed that CBA typically requires less time to perform than RFCA but is associated with an increased likelihood of transient phrenic nerve palsy. The analysis did not show any significant difference between the two methods, and the results indicated similarities in clinical outcomes that included acute PVI, overall complication rates, pericardial effusion, and fluoroscopy time. These insights are valuable for healthcare providers in selecting the most appropriate ablation technique according to individual patient needs.

## Supplementary Information

Additional file 1

## Data Availability

All data generated or analyzed during this study are included in this published article and its supplementary information files.

## Abbreviations

AF: Atrial fibrillation
PVI: Pulmonary vein isolation
RFCA: Radiofrequency catheter ablation
CBA: Cryoballoon ablation
PAF: Paroxysmal atrial fibrillation
U.S: United States
PRISMA: Preferred reporting items for systematic reviews and meta-analyses
RoB 2: Cochrane risk-of-bias tool for randomized trials
RR: Risk ratio
CIs: Confidence intervals
RF: Radiofrequency
LA: Left atrium
