# Supplementary material for: Comparative safety and effectiveness of cryoballoon versus radiofrequency ablation for atrial fibrillation: a systematic review and meta-analysis
Source: Egypt Heart J. 2025 Feb 3;77:18. doi: 10.1186/s43044-025-00611-9 (PMC11790551; doi:10.1186/s43044-025-00611-9)
Supplement: Supplementary file 1 — Additional file 1 [file 43044_2025_611_MOESM1_ESM.docx]

Table of Contents

[Secondary Outcomes: 2](#_Toc145161974)

[Sensitivity Analysis: 8](#_Toc145161975)

[Supplementary Table S1: Search Strategy 13](#_Toc145161976)

[Supplementary Table S2: GRADE 14](#_Toc145161977)

Secondary Outcomes:

***
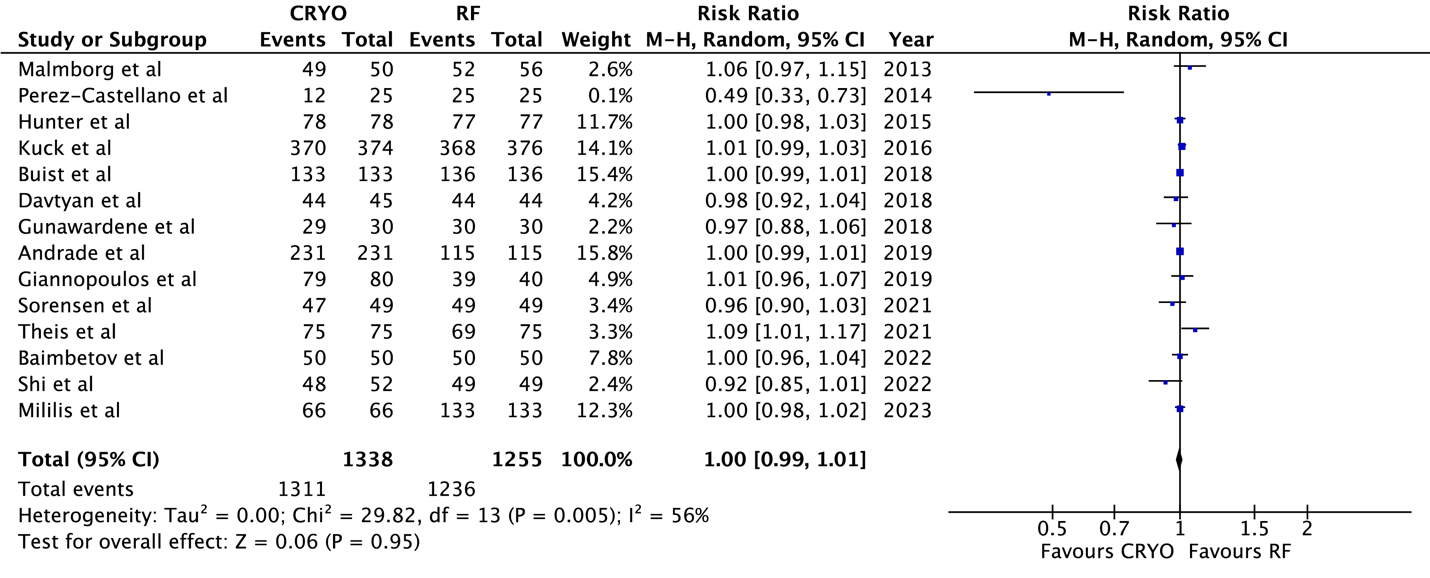
***

Figure S 1: Forest plot for the proportion of acute PVI


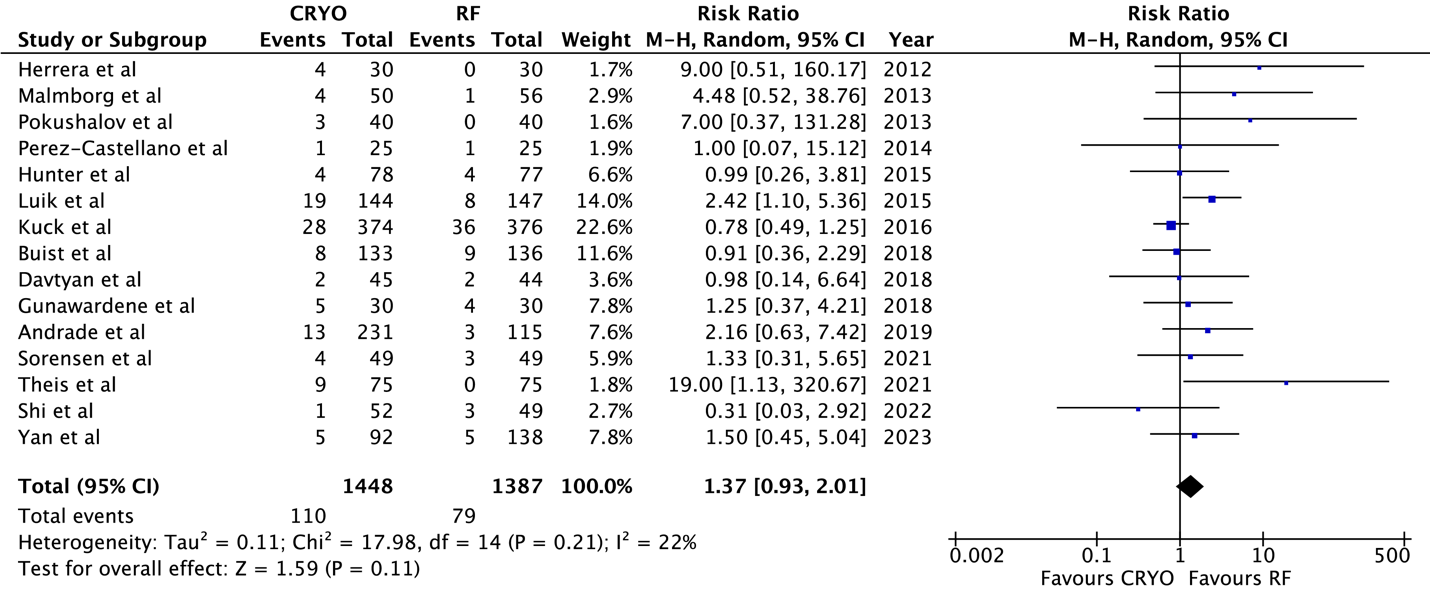


Figure S 2: Forest plot for the proportion of the occurrence of total complications


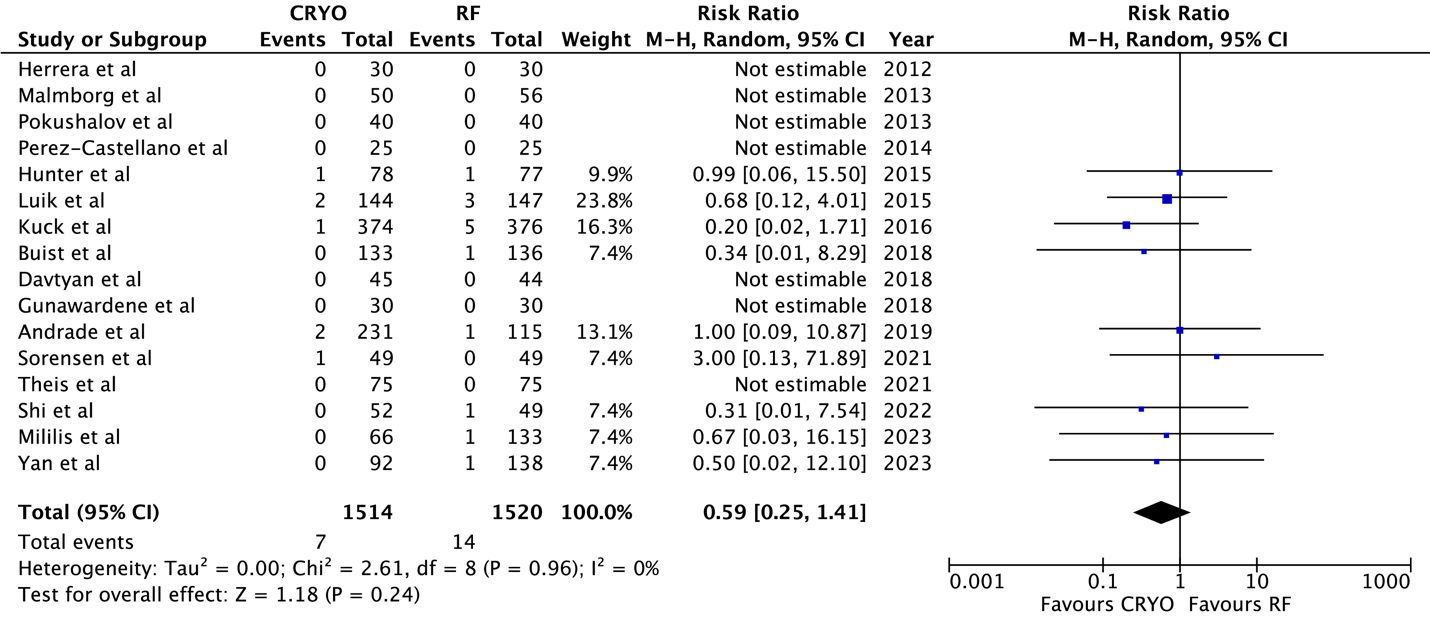


Figure S 3: Forest plot for the proportion of the occurrence of pericardial effusion


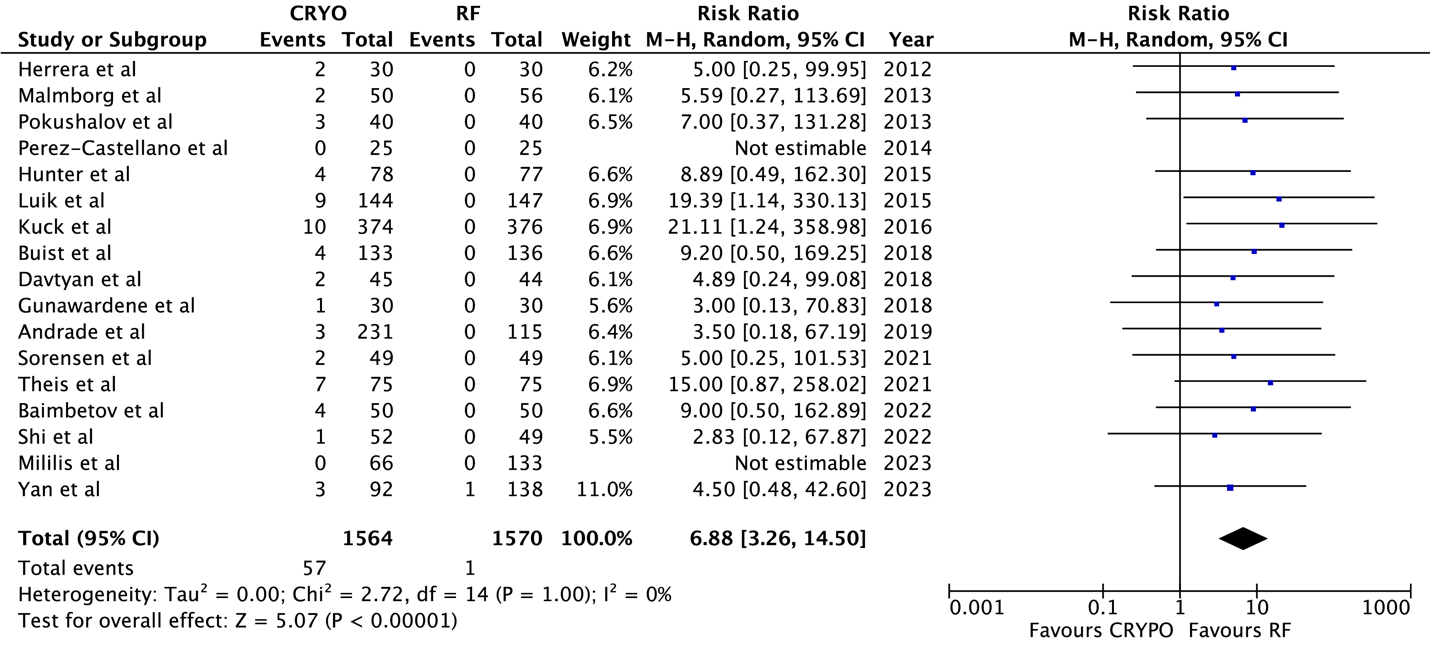


Figure S 4: Forest plot for the proportion of the occurrence of transient phrenic nerve palsy


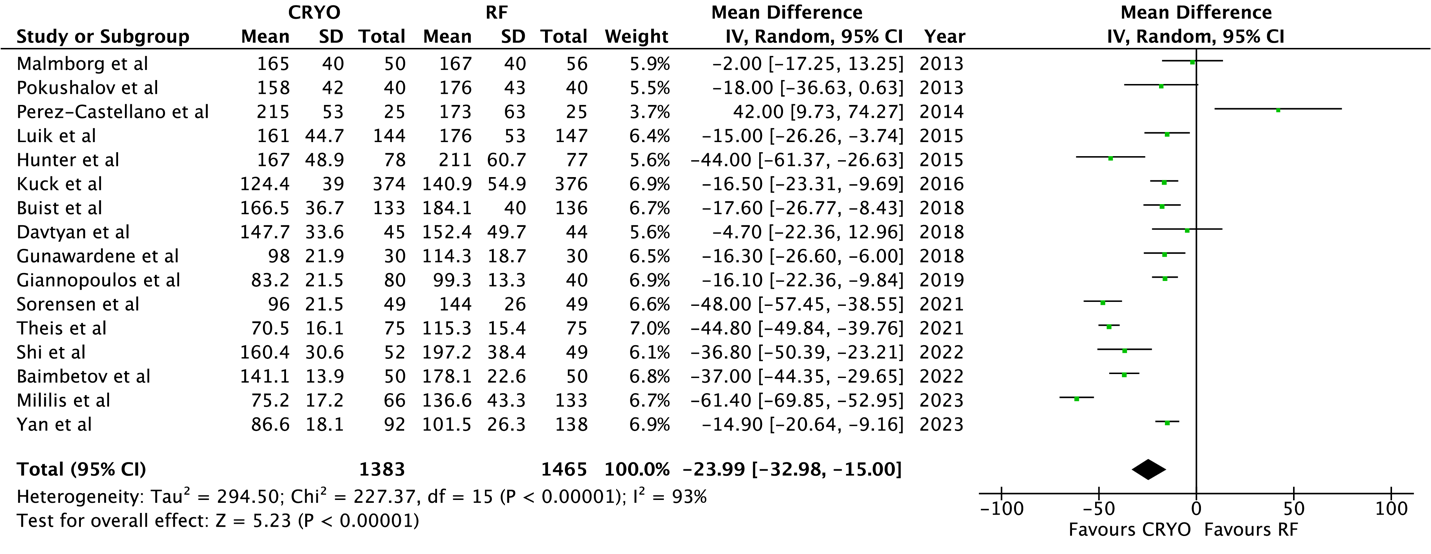


Figure S 5: Forest plot of procedure time


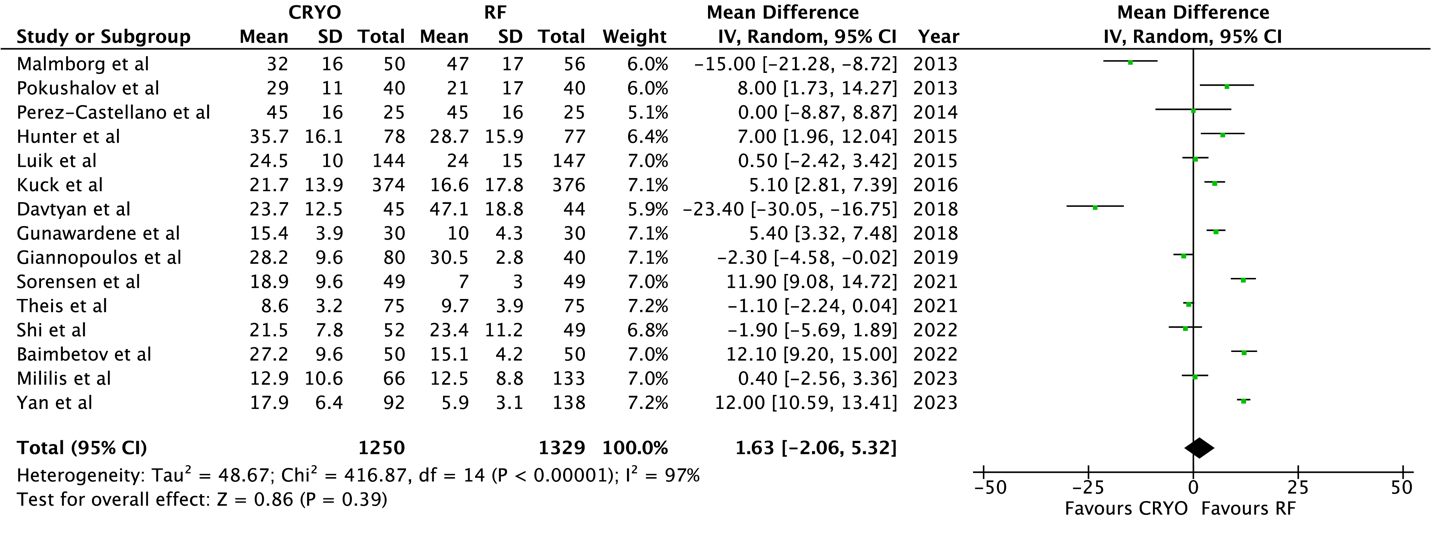


Figure S 6: Forest plot of fluoroscopy time

# Sensitivity Analysis:


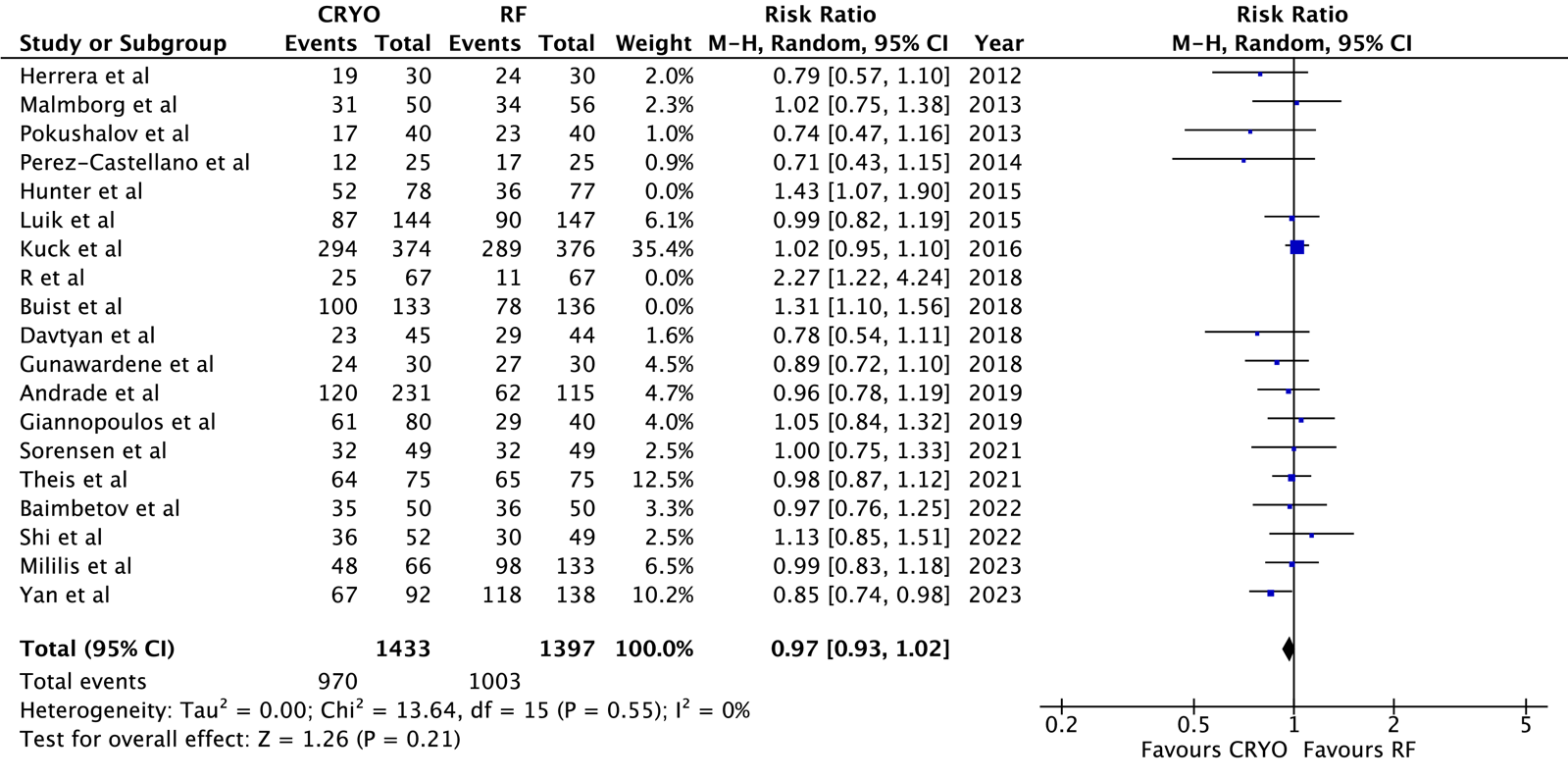


Figure S 7: Forest plot for patients with Atrial Fibrillation after sensitivity analysis


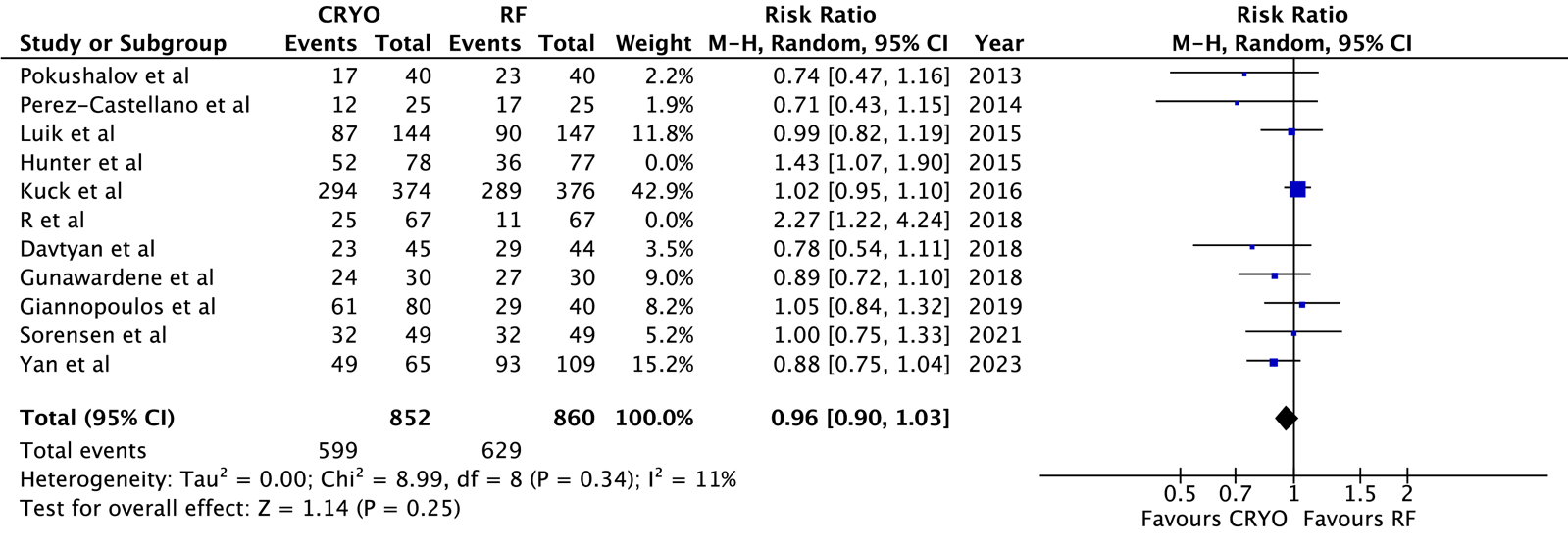


Figure S 8: Forest plot for patients with Paroxysmal Atrial Fibrillation after sensitivity analysis


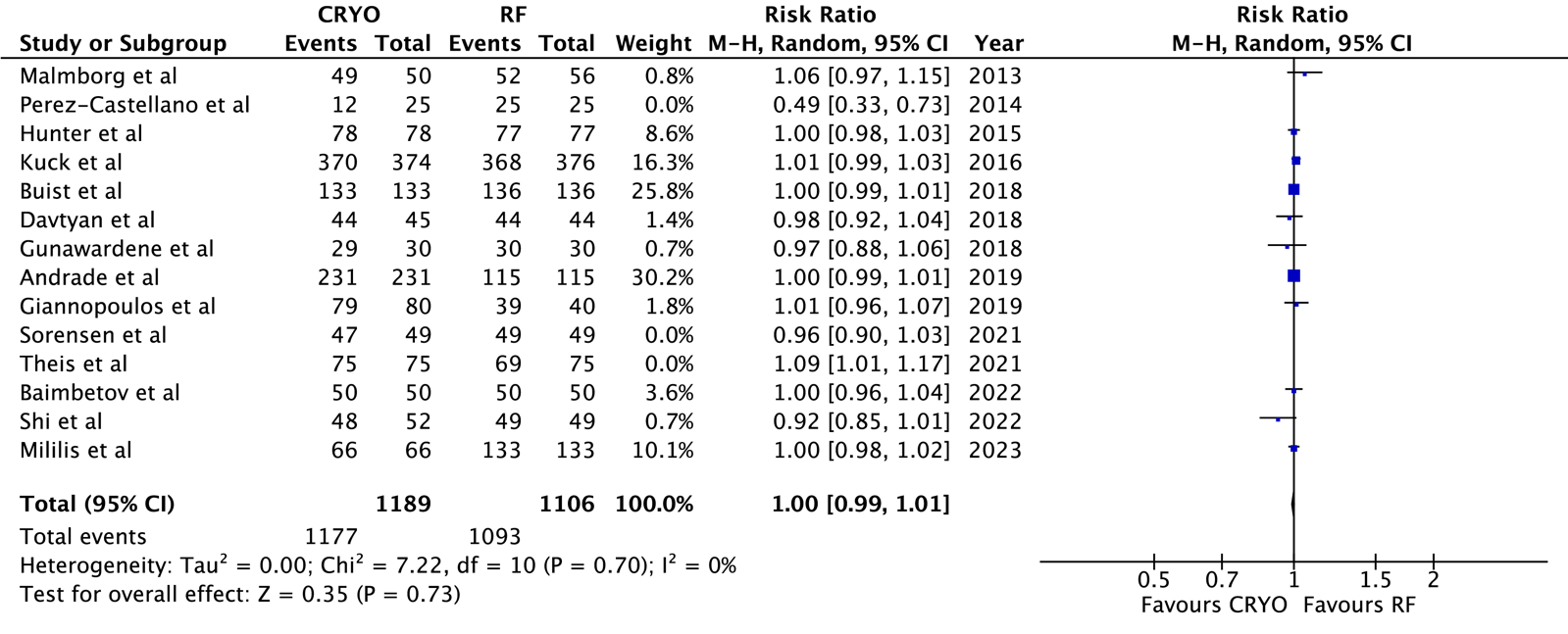


Figure S 9: Forest plot for acute PVI after sensitivity analysis


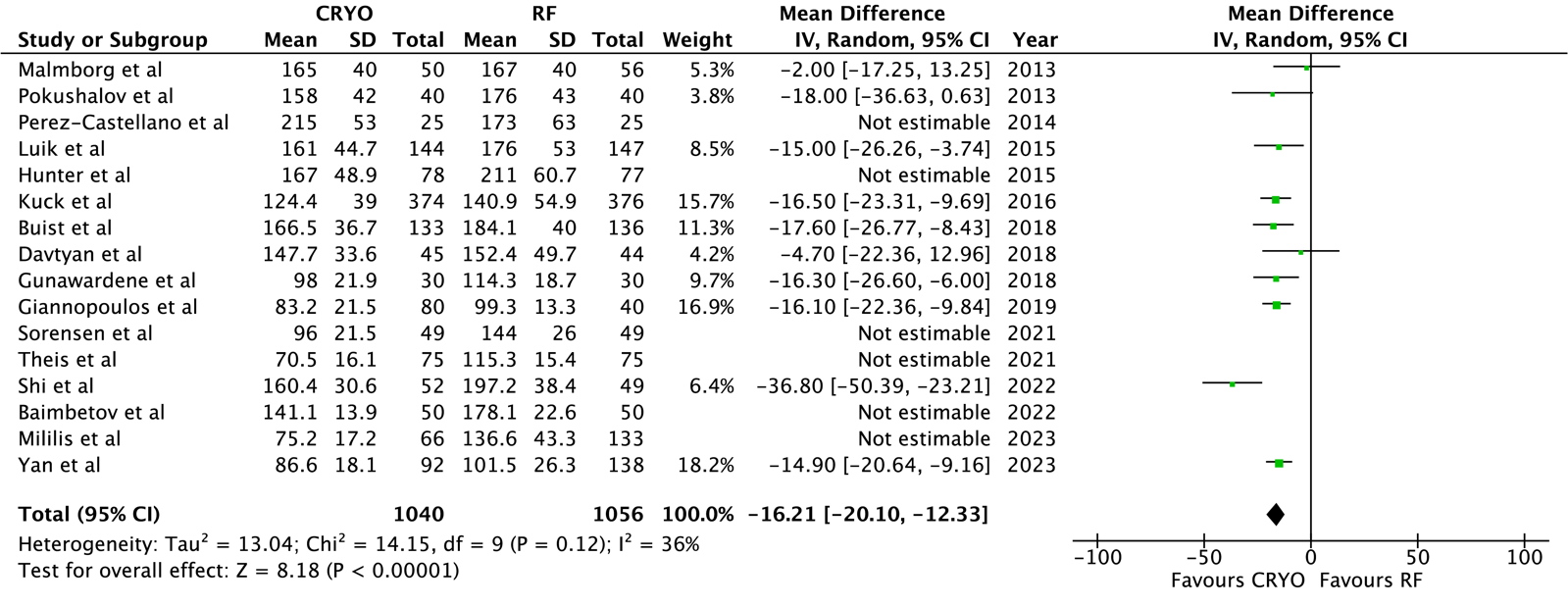


Figure S 10: Forest plot for procedure time after sensitivity analysis


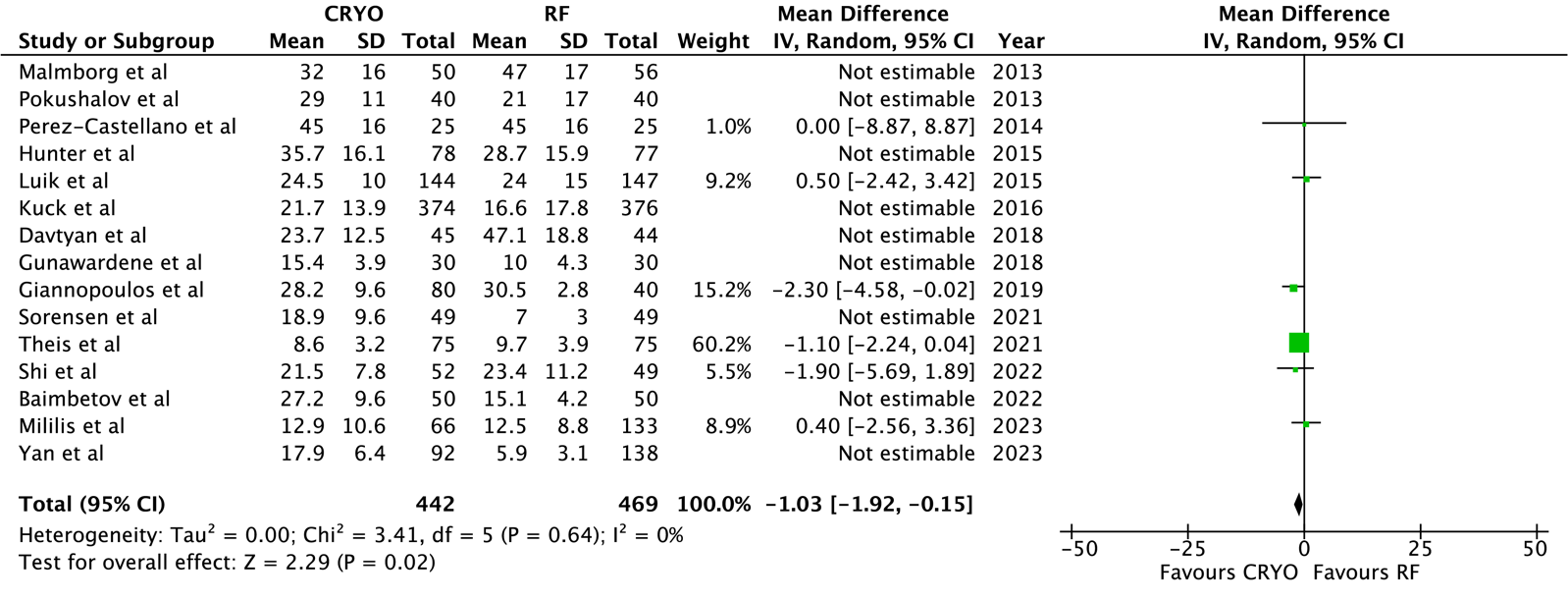


Figure S 11: Forest plot for fluoroscopy time after sensitivity analysis

# Supplementary Table S1: Search Strategy

| **Database** | | **Query** | **Search Details** | **Results** |
| --- | --- | --- | --- | --- |
| PubMed | (Cryoballoon ablation OR cryoballoon catheter ablation OR cryoablation) AND (radiofrequency catheter ablation OR radiofrequency ablation) AND (atrial fibrillation OR paroxysmal atrial fibrillation) | | ((("cryoballoon"[All Fields] OR "cryoballoons"[All Fields]) AND ("ablate"[All Fields] OR "ablated"[All Fields] OR "ablates"[All Fields] OR "ablating"[All Fields] OR "ablation"[All Fields] OR "ablational"[All Fields] OR "ablations"[All Fields])) OR (("cryoballoon"[All Fields] OR "cryoballoons"[All Fields]) AND ("catheter ablation"[MeSH Terms] OR ("catheter"[All Fields] AND "ablation"[All Fields]) OR "catheter ablation"[All Fields])) OR ("cryoablated"[All Fields] OR "cryosurgery"[MeSH Terms] OR "cryosurgery"[All Fields] OR "cryoablation"[All Fields] OR "cryoablations"[All Fields])) AND ("catheter ablation"[MeSH Terms] OR ("catheter"[All Fields] AND "ablation"[All Fields]) OR "catheter ablation"[All Fields] OR ("radiofrequency"[All Fields] AND "catheter"[All Fields] AND "ablation"[All Fields]) OR "radiofrequency catheter ablation"[All Fields] OR ("radiofrequency ablation"[MeSH Terms] OR ("radiofrequency"[All Fields] AND "ablation"[All Fields]) OR "radiofrequency ablation"[All Fields])) AND ("atrial fibrillation"[MeSH Terms] OR ("atrial"[All Fields] AND "fibrillation"[All Fields]) OR "atrial fibrillation"[All Fields] OR ("atrial fibrillation"[MeSH Terms] OR ("atrial"[All Fields] AND "fibrillation"[All Fields]) OR "atrial fibrillation"[All Fields] OR ("paroxysmal"[All Fields] AND "atrial"[All Fields] AND "fibrillation"[All Fields]) OR "paroxysmal atrial fibrillation"[All Fields])) | 1745 |
| Cochrane Library |  | | 1. #1: MeSH descriptor: [Atrial Fibrillation] explode all trees 2. (Atrial Fibrillation Next (Paroxysmal* or Persistent*)):ti,ab,kw (Word variations have been searched) 3. #1 OR #2 4. MeSH descriptor: [Ablation Techniques] explode all trees 5. (Ablation Next (cryoballoon* or radiofrequency*)):ti,ab,kw OR (Radiofrequecny NEXT (ablation* or catheter ablation*)):ti,ab,kw OR (Cryoballoon Next (ablation* or catheter ablation* or cryoblation)):ti,ab,kw (Word variations have been searched) 6. #4 OR #5 7. #3 AND #6 | 1232 |

# Supplementary Table S2: GRADE

| **Outcomes** | **No. of participants**  **(studies)** | **Effect estimate**  **(95% CI)** | **Risk of bias** | **Inconsistency** | **Indirectness** | **Imprecision** | **Publication Bias** | **Quality of Evidence**  **(GRADE)** |
| --- | --- | --- | --- | --- | --- | --- | --- | --- |
| Proportion of patients free from AF in all 19 studies | 3388 | 1.00 (0.93, 1.07) | Not serious | Not serious | Not serious | Serious^1^ | Undetected | ⊕⊕⊕⊝  MODERATE |
| Proportion of patients free from AF (Paroxysmal) in 11 studies | 2001 | 0.99 (0.89, 1.10) | Not serious | Moderate | Not serious | Not serious | Undetected | ⊕⊕⊕⊕  HIGH |
| Proportion of patients of acute PVI | 2593 | 1.00 (0.99, 1.01) | Not serious | Not serious | Not serious | Serious^1^ | Undetected | ⊕⊕⊕⊝  MODERATE |
| Occurrence of total complications | 2835 | 1.37 (0.93, 2.01) | Not serious | Not serious | Not serious | Serious^2^ | Suspected | ⊕⊕⊝⊝  LOW |
| Occurrence of Pericardial Effusion | 3034 | 0.59 (0.25, 1.41) | Not serious | Not serious | Not serious | Serious^3^ | Suspected | ⊕⊕⊝⊝  LOW |
| Occurrence of Transient Phrenic nerve palsy | 3134 | 6.88 (3.26, 14.50) | Not serious | Not serious | Not serious | Serious^4^ | Undetected | ⊕⊕⊕⊝  MODERATE |
| Procedure Time | 2848 | -24.00 (-32.99, -15.01) | Not serious | Serious | Not serious | Serious^5^ | Suspected | ⊕⊝⊝⊝  VERY LOW |
| Fluoroscopy Time | 2579 | 1.63 (-2.06, 5.32) | Not serious | Serious | Not serious | Serious^6^ | Suspected | ⊕⊝⊝⊝  VERY LOW |

^1^ 95% CI fails to exclude important benefit

^2^ Optimal information size (OIS) of 9470 was not met

^3^ Optimal information size (OIS) of 109498 was not met

^4^ Optimal information size (OIS) of 43568 was not met

^5^ Optimal information size (OIS) of 25801 was not met

^6^ Optimal information size (OIS) of 8038 was not met
